# Critical Review of Hearing Rehabilitation in Pediatric Oncology: Specific Considerations and Barriers

**DOI:** 10.3390/curroncol32090509

**Published:** 2025-09-13

**Authors:** Guillaume Courbon, Laurie Lugnier, Johnnie K. Bass, Thomas E. Merchant, Thierry Morlet, Celine Richard

**Affiliations:** 1 Sainbiose U1059, Inserm, University of Saint Etienne, Mines Saint Etienne, 42023 Saint Etienne, France; 2 Department of Rehabilitation Services, St. Jude Children’s Research Hospital, Memphis, TN 38105, USA; 3 Department of Radiation Oncology, St. Jude Children’s Research Hospital, Memphis, TN 38105, USA; 4 School of Communication Sciences and Disorders, University of Memphis, Memphis, TN 38152, USA; 5 Division of Pediatric Otolaryngology, St. Jude Children’s Research Hospital, Memphis, TN 38105, USA; 6 Department of Otolaryngology—Head and Neck Surgery, University of Tennessee Health Science Center, Memphis, TN 38163, USA

**Keywords:** pediatric oncological population, hearing loss, radiotherapy, chemotherapy, hearing rehabilitation, implanted hearing devices

## Abstract

Childhood cancer treatments, including chemotherapy, radiation therapy, and combined modalities, pose significant risks to auditory function due to their ototoxic effects. Cisplatin, a chemotherapeutic agent commonly used in pediatric oncology, causes dose-dependent irreversible sensorineural hearing loss by damaging the inner ear structures, primarily through the generation of reactive oxygen species and the activation of apoptotic pathways. Radiation therapy exacerbates these effects, contributing to both sensorineural and conductive hearing loss via mechanisms such as vascular injury, inflammation, and fibrosis. The severity of hearing loss is influenced by the treatment timing, the cumulative dose, patient age, genetics, and concurrent therapies. The damaging effects of chemotherapy and radiation extend beyond the cochlea, involving the surrounding temporal bone as well as multiple levels of the auditory pathway. While pediatric patients may be candidates for bone-anchored hearing devices or cochlear implants, the need for serial imaging and the potential for implant-related MRI artifacts can complicate the timing of hearing rehabilitation. Moreover, the impact on the subcortical and cortical auditory structures may further influence the rehabilitation outcomes. This scoping review lays the foundation for future clinical and research efforts focused on the development of comprehensive pediatric guidelines for hearing preservation, monitoring, and rehabilitation, while also fostering multidisciplinary collaboration.

## Simple Summary

Cancer treatment in children, such as chemotherapy and radiation, can cause serious permanent hearing loss by damaging the external, middle, and/or the inner ear. Several factors influence how much the hearing is affected at the level of the inner ear, including the child’s age, the total treatment dose, genetic background, and the use of other therapies. The damage can also extend beyond the ear, affecting nearby bones and even the brain areas involved in hearing. While a new medication may help reduce some of the harmful effects of cisplatin-based chemotherapy, it is not suitable for all children, and its benefits are still limited. Some children may benefit from hearing devices such as cochlear implants or bone-anchored hearing aids. However, choosing the right time and type of rehabilitation is complex, especially since these devices can interfere with the MRI scans needed to monitor cancer. More research is needed to guide personalized decisions that balance hearing care with cancer surveillance. This review highlights the urgent need for better strategies to protect hearing during treatment, monitor changes early, and plan effective individualized hearing rehabilitation. It also emphasizes the importance of close collaboration between healthcare providers to support each child’s hearing and overall development.

## 1. Introduction

The continuous progress in innovative therapies, leveraging state-of-the-art technologies, has significantly enhanced the survival rate among the pediatric oncological population [1,2], consequently prompting a shift in focus towards addressing both short- and long-term conditions associated with the disease [3,4,5]. Among these concerns, hearing loss is receiving growing attention. Though not a life-threatening danger, it is a barrier to proper language development [6], cognitive development [7], executive function [8], communication skills, education and social integration [9,10], and overall life quality [11,12,13]. As a result, it is now one of the focus areas for multiple national and international health organizations [14,15,16]. Children with malignancies often face complex factors contributing to hearing impairment, potentially exacerbated by additive effects [17,18].

Interdisciplinary collaboration is essential in managing pediatric cancer patients [19,20]. Advances in multidisciplinary care, preventive strategies, and therapies have significantly improved short- and long-term hearing outcomes [21]. However, oncologic therapies frequently involve the temporal bone, resulting in structural and functional alterations of the auditory system. These changes affect the inner ear and multiple levels of the auditory system [22,23], while also inducing inflammation, infection, and disruption of the temporal bone microarchitecture [24].

This review aims to highlight the multifaceted challenges faced by clinical teams when considering hearing rehabilitation in pediatric oncologic patients and provides a foundation for future research.

## 2. Treatment Modalities for Childhood Cancers and the Pathophysiology of Their Impact on Hearing (Figure 1)

### 2.1. Overview of Ototoxic Treatments in Pediatric Oncology

The most common cancer types affecting children and adolescents include leukemias, brain tumors, and lymphomas [25]. Treatment typically involves surgery, chemotherapy, radiation therapy (RT), or a combination of these modalities [18]. Chemotherapy-induced ototoxicity primarily results in sensorineural hearing loss [17,26], whereas radiation-related ototoxicity may manifest as a sensorineural component, a conductive component, or a mixed presentation [27,28,29].

**Figure 1:**
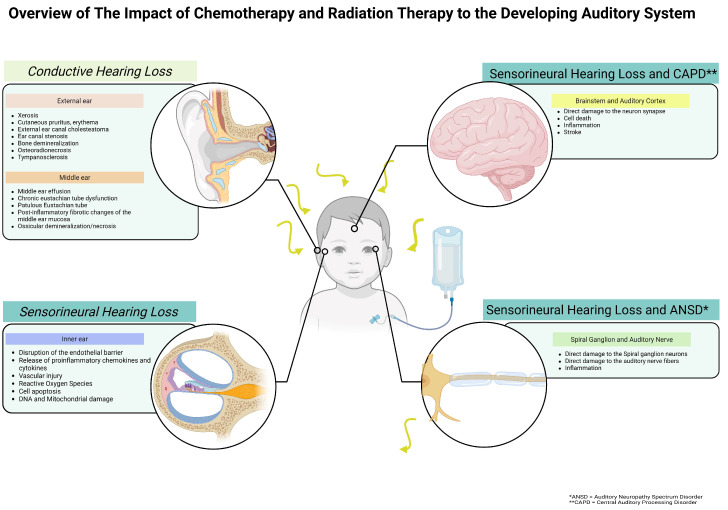
Schematic overview of the impact of chemotherapy and radiation therapy on the developing auditory system.

### 2.2. Cisplatin-Induced Ototoxicity

Among the chemotherapy agents, platinum-based compounds such as cisplatin are frequently employed [30]. In the cochlea, cisplatin undergoes structural transformation in response to low chloride concentrations, resulting in intracellular accumulation and impaired efflux [31,32]. Cisplatin ototoxicity involves multiple mechanisms that induce sustained cellular stress, primarily through the generation of reactive oxygen species (ROS) [33,34] along with a reduction in antioxidant defenses and increased intracellular calcium levels [35,36] among other pathways [37]. Its dose-dependent cytotoxicity primarily targets outer hair cells, spiral ganglion neurons, and the stria vascularis [32,38,39], following a basal-to-apical gradient, with the basal outer hair cells being most vulnerable [40], likely due to the rapid accumulation of high levels of cisplatin in the basal turn scala tympani, with delayed elimination relative to serum [41]. Tonotopic variations in transport mechanisms, such as the copper transporter (CTR1), organic cation transporter 2 (OCT2) and mechanoelectrical transduction (MET) channel complex [42], result in increased cisplatin uptake in basal hair cells, reflecting variations in transporter expression and activity along the cochlea [43]. Additional factors contributing to vulnerability include disrupted calcium homeostasis and lower glutathione levels in basal outer hair cells, heightening their susceptibility to oxidative stress [44,45]. The risk and severity of cisplatin-induced hearing loss are influenced by multiple factors, including the patient’s age (particularly under 5 years) [46,47], cumulative dose (typically 400–500 mg/m^2^ in children) [48], nutritional status, duration of treatment, method of infusion, number of chemotherapy cycles, renal function, and concurrent radiation therapy. This type of ototoxicity typically presents as irreversible, sensorineural, and bilateral, primarily impacting high frequencies [49]. Onset may occur within hours to days after cisplatin administration, with hearing loss potentially progressing over time [50,51], as cisplatin is retained in the cochlea for months to years in both mouse and human cochlea as shown in temporal bone studies [41]. Tinnitus is a commonly reported accompanying symptom [52]. This prolonged retention contributes to progressive cochlear damage by sustaining oxidative stress and inflammation, while impairing mitochondrial function and DNA repair [53,54]. These processes collectively lead to the progressive and irreversible loss of inner ear cells. Interindividual variability in susceptibility is partly genetic. Variants in genes such as thiopurine methyltransferase (TPMT), which influence drug metabolism, have been linked to an increased risk of cisplatin-induced ototoxicity [55].

The impact of chemotherapy, and cisplatin in particular, extends beyond the inner ear. For instance, macrophages and fibroblasts—critical to the wound healing process—are similarly susceptible to its cytotoxic effects, as are cancer cells [56].

### 2.3. Radiation Therapy and Effects on the Inner Ear

The combination of cisplatin and RT is a common therapeutic approach for pediatric cancers [57], enhancing survival but causing ototoxicity, which manifests as hearing loss and tinnitus [49]. Hearing-related side effects are often bilateral, increase with the cumulative dose, tend to worsen over time, and are typically permanent. In contrast to chemotherapy-induced sensorineural damage, radiation therapy can cause conductive hearing loss due to structural and functional changes within the outer and middle ear. These changes may include narrowing of the ear canal, thickening of the eardrum, and impaired function of the middle ear or Eustachian tube, commonly resulting in middle ear effusion [58,59,60]. Among RT-related causes, otitis media with effusion (OME) is the most frequently observed contributor to conductive hearing loss [24,61]. Nevertheless, additional complications, such as chronic middle ear infections, persistent tympanic membrane perforation, post-inflammatory fibrosis within the middle ear, and ossicular demineralization, can also contribute [24]. The radiation-induced effects encompass a complex cascade, involving reactive oxygen species (ROS) production, vascular injury, chronic hypoxia, inflammatory response, and myofibroblast activation leading to fibrosis [62]. Vascular injury is a pivotal element in radiation damage, initiating coagulation pathways, vascular occlusion, tissue ischemia, and chronic hypoxia. This hypoxic state perpetuates ROS production, exacerbating the cellular damage. At the microscopic level, radiation disrupts the endothelial barrier, increasing the vascular permeability, releasing proinflammatory chemokines and cytokines, and facilitating immune cell migration [63,64]. These acute effects, akin to other off-target radiation effects, may lead to long-term vascular dysfunction if unaddressed [64]. Endothelial injury is increasingly attributed to cancer treatment, directly impacting the vasculature by inducing endothelial cell apoptosis and senescence and altering normal vascular homeostasis, contributing to a systemic chronic inflammatory state. Non-irradiated tissues are influenced by signals from nearby radiated cells through the bystander effect [65]. Early radiation-induced damage to the ear is visible on a macroscopic level as swelling, inflammation, and scaling of the tissues in the outer, middle, and inner ear [66]. When the external auditory canal is exposed to radiation, there is increased vulnerability to infections of the soft tissues in that area [67]. Within the inner ear level, the stria vascularis is pivotal in mediating inflammation by recruiting macrophages to the damaged area [68,69]. The pediatric population, especially those patients receiving treatment for malignant conditions, face increased risks for chronic OME due to factors such as upper airway infections and eustachian tube alterations [58], leading to significant morbidity. Managing chronic otitis media with effusion in oncologic patients and addressing long-term sequelae through corrective surgery demands a thorough understanding of the complex underlying pathophysiology to optimize the timing while balancing the benefits and risks [70,71]. While temporal bone osteoradionecrosis is a long-term complication observed in the adult population [72], the oncologic population may present with altered temporal bone function at the microstructural level. Ionizing radiation disrupts intracellular homeostasis by directly damaging the DNA and indirectly generating free radicals from water molecules.

### 2.4. Risk Mitigation and Prevention

Recently, proton beam radiotherapy (PBT) has seen exponential growth, especially in managing childhood cancer. PBT, available at select institutions, is globally acknowledged for use in pediatric brain, head, and neck and adult skull base malignancies. It has the potential for superior outcomes in terms of local control while maintaining acceptable levels of toxicity, when compared to photon therapy, in settings where high doses are required at sites adjacent to sensitive normal tissues [73]. Where dose escalation is not required, PBT demonstrates a superior ability to reduce the doses to normal tissues, thereby potentially mitigating both acute and late toxicities [73,74,75,76].

Recent studies suggest that children are more vulnerable to hearing loss than adults, with a correlation between the average radiation dose received by the cochlea and the risk of auditory impairment [28]. Nonetheless, data in children confirming the suggested cochlear radiation limit of 30 to 45 Gy, along with a clearer understanding of how cisplatin and radiation therapy interact to worsen hearing loss, are still scarce [77,78].

Hearing loss induced by chemotherapy and RT may be further exacerbated by the potential additive effect of a bone marrow transplant [26], heightening the risk for patients undergoing multimodal therapy with combined cisplatin and RT administration [27,47,79,80], Individuals with central nervous system (CNS) tumors face heightened risks of hearing loss from additional factors, including exposure to the neurotoxic vinca-alkaloid vincristine, cerebrospinal fluid-shunt implants, and brain surgeries involving the auditory system [81,82,83]. The effects of the therapies may also extend to neural structures at the nerve level or higher, including cortical areas [84,85], emphasizing that the prevention of ototoxic hearing loss requires the implementation of primary, secondary, and tertiary strategies [86]. Primary prevention aims to avert the onset of hearing loss, often through modifying treatments or exploring alternative pharmaceutical approaches [87]. Pharmacologic otoprotectants show promise in clinical trials; however, challenges remain regarding regulatory approval and global accessibility [88,89,90]. Sodium thiosulfate, is currently FDA-approved to reduce the risk of cisplatin-related SNHL in children aged 1 month to 18 years with localized non-metastatic solid tumors [91]. Despite this, 30% to 40% of children receiving cisplatin still develop SNHL, even when treated with sodium thiosulfate [92].

Ultimately, while auditory symptoms may represent a common clinical endpoint of cancer therapy, the underlying mechanisms vary based on the treatment type and the specific tissues affected. A deeper understanding of these complex pathophysiological processes is essential to minimize long-term auditory complications in childhood cancer survivors.

## 3. Effects of Oncologic Therapies on the Temporal Bone: Insights into Bone Mineral Density and Candidate Biomarkers of Temporal Bone Metabolisms in Pediatric Patients

Early research has identified decreased bone mineral density (BMD) in the spine and long bones of pediatric survivors who underwent cranial RT for conditions such as medulloblastoma [93], as well as acute lymphoblastic leukemia (ALL) [94], despite these skeletal sites not being directly in the high-dose radiation field [95,96]. The reported incidence of osteopenia in childhood cancer survivors ranges from 33% to 51% [97], with cranial irradiation during childhood consistently identified as the strongest predictor of low BMD in adulthood [98]. Similar findings have been observed in other long-term survivor cohorts [99]. However, conventional BMD assessments, such as lumbar spine BMD, femoral BMD, and total body BMD do not effectively capture the localized damage to the temporal bone. Currently, no standardized method exists for directly evaluating the mineralization of the temporal bone. Similarly, indirect assessment using computed tomography (CT) is limited by concerns over radiation exposure, which restricts the routine use of CT imaging in pediatric populations [100].

In the context of surgical strategies involving implantable devices, the quality of bone and, particularly, its mineral density and microarchitecture, is a critical factor influencing surgical outcomes and long-term device integration [101,102]. While the literature supporting this relationship is well-established in the field of dental implants [103,104], corresponding data on hearing implants would benefit from additional research [24,105,106,107]. Nonetheless, the principles of osseointegration and bone health are likely to be similar. Given the current gap in research and the limited access to direct evaluation methods for temporal bone mineralization in children, circulating biomarkers of bone metabolism present a promising alternative for assessing bone health [108]. These biomarkers offer valuable insights into various aspects of bone turnover, including formation, resorption, and the dynamic balance between the two. They are well-established proxies, used in clinics, for bone cell activity [109,110,111,112].

Other therapies commonly included in pediatric cancer treatment regimens, such as cisplatin and corticosteroids are well-known potent inducers of osteopenia [113,114,115]. Consequently, the combined effects of the tumor itself, radiotherapy, and these pharmacologic agents may synergistically contribute to compromised bone health [116].

Enhanced detection of alterations in bone mineralization and microarchitecture, at both molecular and structural levels, hold significant implications for hearing rehabilitation strategies. Biomarkers capable of identifying early changes in the temporal bone quality could enable timely intervention and help anticipate potential challenges related to the placement and long-term stability of surgical hearing devices.

## 4. Types of Hearing Implants and Considerations for Implant Osseointegration (Figure 2)

Early and individualized hearing rehabilitation is best achieved through a coordinated effort between oncologists, radiation therapists, audiologists, and otolaryngologists. Rehabilitation options may include standard hearing aids, CROS and bi-CROS devices, bone conduction systems, including osseointegrated implants, as well as non-surgical alternatives such as bone conduction devices on soft bands, eyeglass-mounted aids, adhesives, or cochlear implants when indicated.

**Figure 2:**
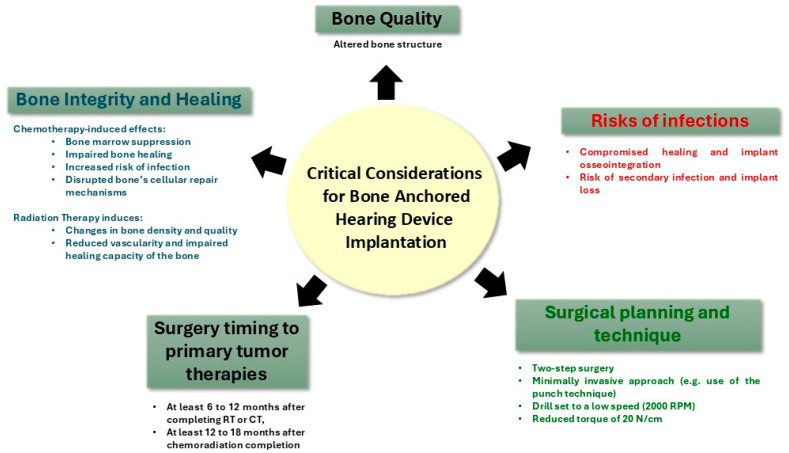
The effects of therapeutic interventions on the temporal bone and its implications for the placement of bone-anchored hearing devices.

### 4.1. Bone Conduction Hearing Devices: Indications and Options

When planning hearing rehabilitation with osseointegrated implants in pediatric oncology patients, surgical challenges and compromised bone quality due to cancer treatments must be carefully considered [107]. Medications such as cyclosporine A, methotrexate, cisplatin, proton pump inhibitors, anticonvulsants, selective serotonin reuptake inhibitors, warfarin, and specific types of heparins might impede the osseointegration process [117]. Cisplatin, in particular, has been shown to hinder bone regeneration around implants in animal models, compromising the titanium integration of dental implants [118,119]. However, long-term outcomes suggest that implant survival remains unaffected when placement occurs approximately 10.5 months after chemotherapy [120].

Bone conduction [121] hearing devices (BCHD) are effective for conductive or mixed hearing loss and single-sided deafness, where sound is transmitted to the functioning contralateral cochlea [122,123]. These devices are available in both surgical (osseointegrated) and non-surgical forms, including soft bands, metal head bands, eyeglasses, or adhesive. While non-surgical devices are suitable for younger children, they offer reduced sound transmission, especially at higher frequencies, due to soft tissue attenuation [124]. Additionally, children over age six often prefer a surgical option due to peer-related cosmetic concerns [125]. BCHDs are particularly beneficial in cases of a conductive component ≥30 dB or when traditional hearing aids are not tolerated.

Surgically implanted BCHDs are either percutaneous or transcutaneous [126]. Percutaneous devices establish a direct coupling between the transducer and the skull bone via a percutaneous abutment, eliminating skin or soft tissue attenuation. In contrast, transcutaneous devices, whether passive or active, transmit vibrations either directly through an implanted subcutaneous portion or via transcutaneous electromagnetic signals, with passive systems exhibiting some degree of soft tissue attenuation [127]. The FDA has approved the surgical implantation of BCHDs in children aged 5 and older [128].

As part of pre-surgical planning, CT imaging of the temporal bone is primarily used to assess bone thickness and guide optimal implant placement [129,130]. However, its ability to evaluate bone quality is limited. Cone-beam CT has shown promise in the pre-surgical assessment of bone quality for dental implants [131,132]; however, its utility in the context of BCHD has yet to be clearly established [133,134]. Ideally, techniques such as microCT could provide high-resolution insights into bone architecture [135,136], but their use in vivo for temporal bone evaluation is not feasible. Consequently, intraoperative evaluation remains essential. The recent introduction of dual-energy CT, which enables assessment of bone mineral density by analyzing calcium and other mineral content [137,138], offers a promising advancement. This technique may hold future value in preoperative planning for BCHD placement.

When considering a percutaneous device, a two-stage minimally invasive punch technique is advised based on our pediatric experience to minimize tissue trauma and improve osseointegration. The literature also supports low-speed drilling and a torque of 20 N/cm to minimize tissue trauma, preserve bone quality, and enhance osseointegration [24]. While these devices produce fewer magnetic resonance imaging (MRI) artifacts, they carry risks such as osseointegration failure, implant extrusion, and soft tissue complications, including adverse skin reactions and soft tissue infections, observed in up to 84% of patients [127].

Transcutaneous BCHDs can be passive or active systems. Passive transcutaneous systems aim to overcome the limitations of percutaneous implants. While they avoid skin-penetrating components and reduce cosmetic concerns [139,140], they may lead to sound attenuation [141]. Conversely, active transcutaneous devices aim to optimize the advantages of both percutaneous and passive transcutaneous implants, avoiding complications related to skin and soft tissue while minimizing sound attenuation through soft tissue [127]. In single-sided deafness, modern active transcutaneous devices improve high-frequency hearing and speech understanding in noisy environments [142]. Although these implants have shown benefits in the general pediatric population, their use among pediatric oncology patients remains limited. Several factors may contribute to this, one of which is the significant MRI artifact they produce, potentially interfering with imaging-based surveillance of the brain [143].

### 4.2. Cochlear Implants in Pediatric Oncology

In pediatric patients experiencing severe to profound SNHL and who derive limited improvement from conventional hearing aids, cochlear implantation is recommended as the next-line intervention to help reduce further challenges related to their hearing impairment [11]. Any level of hearing loss has the potential to hinder language development, verbal proficiency, and reasoning skills [144]. A prevailing concern in the general population is the prevalence of OME, a common middle ear infection in childhood. Nevertheless, studies in typical cases suggest that cochlear implantation is generally safe in children with OME, implying that deferring the implantation process may be unnecessary [145]. While one might argue that oncology patients, facing an elevated risk of eustachian tube dysfunction and alterations in the middle ear mucosa and function, encounter additional challenges, recent reports indicate that these factors do not seem to impede eligibility for cochlear implantation in the pediatric oncologic population [146]. Furthermore, recent studies endorse electric–acoustic stimulation, utilizing two technologies—a cochlear implant plus acoustic amplification—to cover the full range of hearing, as a viable treatment option for this specific population [147].

When planning surgery, several factors require attention: radiation-induced inflammation and fibrosis in the middle ear can hinder dissection, while radiation-related demineralization calls for extra caution during drilling of the facial recess. Scar tissue and cochlear sclerosis can make electrode insertion difficult [24]. Additionally, the risk of wound dehiscence and infection is increased [56]. While MRI alone may suffice in the general population [148] combined CT and MRI offer complementary insights essential for surgical planning in the pediatric oncologic population. CT delineated bony anatomy; MRI detects early tissue changes such as early fibrosis, and this dual-modality approach is standard in our oncologic cohort.

### 4.3. MRI-Related Considerations for Hearing Implants

Advances in cochlear implant technology now allow many patients to safely undergo MRI, provided specific safety guidelines are followed [149]. Similar observations apply to bone conduction implants, wherein the magnet remains securely in place, signifying a noteworthy enhancement [150]. This development opens the possibility of considering these implants for use in the pediatric oncology population, where serial follow-up MRI is needed. The use of implanted hearing devices is increasing, and many patients with these devices need MRI for clinical reasons. However, both cochlear and transcutaneous bone conduction implants create MRI artifacts due to the internal magnet and differences in magnetic susceptibility between the implants and adjacent soft tissues [151]. MRI compatibility with otological implants has both clinical and practical implications. In the context of cochlear implantation for oncologic recipients, individuals with primary brain tumors undergoing MRI follow-up present a specific challenge owing to the proximity of the field of interest to the cochlear implant, compounded by the smaller anatomy of the pediatric skull [151,152]. Given that CI shadow artifacts can extend approximately 5 to 6 cm, optimizing the MRI sequences is essential. Diffusion-weighted imaging sequences tend to exhibit greater susceptibility to artifact generation compared to other MRI sequences. Fast spin echo and turbo spin echo demonstrate markedly reduced artifact burden, and advanced fat suppression techniques show improvement over traditional fat saturation sequences [151]. Previous studies have shown that the artifact size depends on the implant characteristics, MRI magnet strength, and patient-specific factors [143,151]. However, further research is needed.

Percutaneous BCHDs produce smaller artifacts compared to transcutaneous devices; however, they still produce artifacts near the titanium implant measuring 15.1 to 17.4 mm [152]. Transcutaneous implants generate larger and more variable artifacts depending on the device [143].

## 5. Impact of Chemotherapy, Radiotherapy, and Chemoradiation on the Auditory System

Despite the fact that a significant number of children are eligible for implanted hearing devices, relatively few proceed with implantation [153]. A frequently overlooked factor is the impact of chemotherapy and radiation on the auditory system. The number of pediatric CI recipients remains limited, and patients show inconsistent preoperative hearing aid use. Chemotherapy-induced ototoxicity primarily targets cochlear hair cells, but the damage extends beyond the cochlea to the spiral ganglion [154], auditory nerve, and central processing areas, potentially affecting cortical structures both structurally and functionally [41,155,156]. Recent investigations of the function of the brainstem auditory pathways of children with acute lymphoid leukemia submitted to chemotherapy revealed abnormal brainstem and central auditory evoked potentials in some children, with a predominance of impaired auditory pathways in the lower brainstem [22,157]. These results suggest that changes observed in the brainstem- and central auditory-evoked potentials are due to the neurotoxicity of certain drugs, rather than disease progression, since examination of the children’s cerebrospinal fluid were negative for neoplastic cells, indicating that the disease had not infiltrated into the central nervous system. Similarly, the presence of ABR abnormalities in children treated with the chemotherapeutic drug methotrexate suggests a neurotoxic effect of chemotherapy on the central auditory system. Although the mechanism(s) by which methotrexate causes neurotoxicity is(are) not fully understood, its potential neurotoxicity in the central auditory nervous system must be considered [22,23].

Similarly, while animal and human studies on irradiated ears showed damage to various cochlear structures (membranous labyrinth, hair cells, and stria vascularis), they also revealed atrophy of the spiral ganglion cells and the cochlear nerve with radiation alone or in combination with chemotherapy [66,158,159].

Although it has been suggested that the cochlea is more sensitive to the effects of radiation than the brain or auditory nerves, radiation-induced ear pathology is not uncommon and can affect all parts of the auditory system [160,161,162,163].

Although neurons may exhibit some degree of resistance to radiation effects owing to their low mitotic activity, the surrounding neural tissue remains vulnerable due to radiation-induced damage to connective tissue cells that contribute to myelin production and other supportive functions [164]. Evaluations of cranial irradiation on the hippocampus and cortical neurons revealed dose-dependent damage to the synapses similar to changes found in neurodegenerative diseases [165,166,167]. Therefore, irradiation-induced damage to neural components in the auditory system should not be overlooked. Studies using animal models have demonstrated that high doses of radiation led to the destruction of eight nerve fibers [160]. Reports of abnormal auditory brainstem responses in patients diagnosed with radiation induced SNHL have been made [168,169], as have auditory brainstem responses in patients after radiation-therapy for nasopharyngeal carcinoma [170]. Additionally, evidence indicates that damage to the retrocochlear pathways may go undetected in some patients, implying that the true impact of radiation therapy on hearing could be underestimated [171]. Furthermore, a recent animal study suggests that cochlear ribbon synapses may also be a subcellular target of radiation-induced hearing loss, potentially disrupting neural synchrony throughout the auditory pathways [172].

In summary, all patients with post-irradiation SNHL should be evaluated by MRI involving the inner ear as well as the auditory pathways and include auditory-evoked potential testing (both at the brainstem and central level) to objectively evaluate the function of the acoustic nerve and the central auditory pathways.

## 6. Potential Future Directions for Research

Future research endeavors may follow several promising avenues, spanning prevention, early detection, and optimization of rehabilitation strategies related to the auditory system’s chemo- and/or RT-induced injuries. A critical area of investigation involves the development of advanced monitoring strategies through the identification and validation of inner ear-specific [173,174] biomarkers, detectable in peripheral blood samples [175]. While initial progress has been made in this domain, the relationship between cochlear and auditory nerve injury and systemic bone metabolism remains largely unexplored. Further research is needed to elucidate how radiation-induced inner ear damage is reflected in circulating bone turnover markers. Preliminary research indicates that children treated with cranial radiation therapy for diseases such as acute lymphoblastic leukemia and medulloblastoma may experience reduction in bone mineral density, particularly in the spine and long bone [93,94]. Building on these findings, future research could explore potential correlations between hearing loss and bone turnover markers. Such potential biomarkers coupled with inner-ear specific markers could provide a powerful tool for early diagnosis and longitudinal monitoring of cochlear degeneration and radiation-associated auditory dysfunction.

In addition to molecular biomarkers, functional monitoring should incorporate electrophysiological testing to assess the integrity of the auditory pathway. Techniques such as otoacoustic emissions, auditory brainstem response testing, middle latency responses, and cortical auditory evoked potentials, particularly when elicited using varied and complex stimuli [155], can detect early changes in auditory processing and speech perception [176], expanding the early detection of changes in hearing and speech processing [177].

Keeping in mind the ongoing maturation of the key components of the auditory system in children [178,179,180], longitudinal evaluation of neuroplastic changes at both subcortical and cortical levels remains essential. In this context, developing protocols involving electrophysiological functional markers as exemplified in the general literature [181] would offer valuable tools for assessing auditory function across multiple levels of the auditory pathway [156,178,182]. These markers not only would provide valuable insight into localizing the site of impairment and identifying early signs of damage but also would serve as objective indicators of functional recovery and the effectiveness of interventions such as amplification or implanted hearing devices [183,184].

Different avenues remain to be explored in the prevention of hearing loss. While significant progress has been made, particularly with the FDA approval of sodium thiosulfate (STS) [91] as an otoprotective agent in the pediatric oncologic population, indications are still limited. This is largely due to concerns about the potential effect of STS on the antitumor efficacy of cisplatin. Although evidence suggests that delayed administration of STS does not compromise the cisplatin’s oncologic effectiveness [185,186], current protocols rely primarily on intravenous delivery of STS [187]. A new era in otoprotection is now emerging within the otolaryngology community, with growing interest in targeted inner ear drug delivery [87]. The development of new vectors and alternative delivery routes [188,189] has opened promising avenues for localized therapy, potentially enhancing therapeutic precision while minimizing systemic toxicity [190].

Understanding these alterations along the auditory pathway could significantly inform both prognostic modeling and support the development of personalized rehabilitation protocols. However, further progress is needed to address imaging artifacts caused by the magnetic component of implanted hearing devices [151,191].

Advancing our understanding of auditory pathway alterations will be crucial for improving early detection, guiding personalized rehabilitation strategies, and refining preventive approaches, ultimately paving the way for more effective individualized care in the pediatric oncologic population.

## 7. Conclusions

Childhood cancer treatments occur during a critical window of hearing development, where the ototoxic effects of chemotherapy and radiation can cause irreversible damage to multiple levels of the auditory system. Hearing rehabilitation is further complicated by the timing of the primary tumor treatment, compromised bone quality affecting implant integration, and the need for ongoing imaging surveillance that can be hindered by device-related artifacts. These challenges underscore the need for comprehensive standardized guidelines to guide monitoring, prevention, and tailored rehabilitation strategies in pediatric oncologic patients.

## Abbreviations

QoL	quality of life
RT	radiation therapy
OME	otitis media with effusion
CNS	central nervous system
ALL	acute lymphoblastic leukemia
BMD	bone mineral density
LS	lumbar spine
DXA	dual energy x-ray absorptiometry
CS	corticosteroids
PBT	proton beam radiotherapy
ROS	reactive oxygen species
CI	cochlear implant
BCHD	bone conduction hearing devices
MRI	magnetic resonance imaging
